# Beyond Standard Shocks: A Critical Review of Alternative Defibrillation Strategies in Refractory Ventricular Fibrillation

**DOI:** 10.3390/jcm14145016

**Published:** 2025-07-15

**Authors:** Benedetta Perna, Matteo Guarino, Roberto De Fazio, Ludovica Esposito, Andrea Portoraro, Federica Rossin, Michele Domenico Spampinato, Roberto De Giorgio

**Affiliations:** 1 Department of Biomedical and Neuromotor Sciences, University of Bologna, 40126 Bologna, Italy; benedetta.perna@studio.unibo.it; 2 Department of Translational Medicine, University of Ferrara, 44124 Ferrara, Italy; grnmtt@unife.it (M.G.); ludovica.esposito@unife.it (L.E.); andrea.portoraro@unife.it (A.P.); spmmhl@unife.it (M.D.S.); 3 Emergency Department, Sant’Anna University Hospital of Ferrara, 44124 Ferrara, Italy; 4 Emergency Department, Degli Infermi Hospital of Faenza, Local Health Authority of Romagna, 48018 Faenza, Italy; roberto.defazio@unife.it; 5 Emergency Medical Service (SUEM 118), Local Health Authority ULSS5 Polesana, 45100 Rovigo, Italy; federica.rossin@unife.it; 6 St. Annunziata Hospital of Cento, 44042 Ferrara, Italy

**Keywords:** alternative defibrillation strategies, cardiac arrest, double sequential external defibrillation, refractory ventricular fibrillation, resuscitation, vector-change defibrillation

## Abstract

**Background**: Refractory ventricular fibrillation (RVF) is a life-threatening condition characterized by the persistence of ventricular fibrillation despite multiple defibrillation attempts. It represents a critical challenge in out-of-hospital cardiac arrest management, with poor survival outcomes and limited guidance from current resuscitation guidelines. In recent years, alternative defibrillation strategies (ADSs), including dual sequential external defibrillation (DSED) and vector change defibrillation (VCD), have emerged as potential interventions to improve defibrillation success and patient outcomes. However, their clinical utility remains debated due to heterogeneous evidence and limited high-quality data. **Methods**: This narrative review explores the current landscape of ADSs in patients with RVF. MEDLINE, Google Scholar, the World Health Organization, LitCovid NLM, EMBASE, CINAHL Plus, and the Cochrane Library were examined from their inception to April 2025. **Results**: The available literature is dominated by retrospective studies and case series, with only one randomized controlled trial (DOSE-VF). This trial demonstrated improved survival to hospital discharge with ADSs compared to standard defibrillation. DSED was associated with higher rates of return of spontaneous circulation and favorable neurological outcomes. However, subsequent meta-analyses have produced inconsistent results, largely due to the heterogeneity of the included studies. The absence of sex-, gender-, and ethnicity-specific analyses further limits the generalizability of the findings. In addition, practical barriers, such as equipment availability, pose significant challenges to implementation. **Conclusions**: ADSs represent a promising yet still-evolving approach to the management of RVF, with DSED showing the most consistent signal of benefit. Further high-quality research is required to enhance generalizability and generate more definitive, high-level evidence.

## 1. Introduction

Refractory ventricular fibrillation (RVF) is a critical scenario for emergency physicians, often representing a turning point in resuscitation attempts. There is no agreed definition, but it has been operationally outlined as ventricular fibrillation (VF) or pulseless ventricular tachycardia (pVT) still detectable after three consecutive rhythm analyses and standard defibrillations (SDs) separated by 2 min [1]. However, many patients initially respond to defibrillation but subsequently revert to VF or pVT, categorizing them as cases of recurrent VF rather than true RVF [2,3]. Due to this distinction, the true incidence of RVF remains uncertain. Existing data suggest that approximately 50% of patients presenting with a shockable rhythm require more than two defibrillation attempts [4]. However, when this differentiation is applied, true RVF appears to be relatively infrequent, with estimates indicating that fewer than 5% of patients fulfill its definition [5]. In patients with RVF, neither additional defibrillation nor antiarrhythmic drugs have been definitively shown to improve survival to hospital discharge or neurologically intact survival [6,7]. Therefore, alternative defibrillation strategies (ADSs), such as double sequential external defibrillation (DSED) and vector-change defibrillation (VCD), have been proposed, though evidence supporting their effectiveness remains limited. Indeed, the latest guidelines proposed by the American Heart Association (AHA) stated that the usefulness of DSED for refractory shockable rhythms has not been established [8]. Instead, the latest European Resuscitation Council (ERC) guidelines from 2021 did not provide a definitive recommendation for a specific treatment of RVF, suggesting the consideration of VCD, while advising against double sequential external defibrillation (DSED) with a weak recommendation based on low-certainty evidence [9]. This position was justified by a lack of well-designed studies until November 2022, when Cheskes et al. published the first randomized controlled trial (RCT) on the use of these alternative strategies compared with the standard technique [1].

While the findings were promising, the widespread adoption of these strategies has been hindered by inconsistent results and methodological limitations in much of the existing evidence. This narrative review aims to offer a critical appraisal of the current literature, examine definitions, mechanisms, and practical applications of ADSs, discuss the challenges and limitations of their implementation in real-world scenarios, and offer recommendations for future research to address evidence gaps and improve the integration of ADSs into resuscitation protocols.

## 2. Search Strategy

A comprehensive literature search was conducted across multiple databases, including MEDLINE, Google Scholar, the World Health Organization (WHO) repository, LitCovid NLM, EMBASE, CINAHL Plus, and the Cochrane Library, covering publications from their inception to April 2025. A combination of subject headings and keywords relevant to ADSs and RVF were combined using appropriate Boolean operators. A detailed description of the search strategy can be found in the Supplementary Materials (see Supplementary Table S1). Furthermore, relevant papers were hand-retrieved to identify further studies that might have been missed by the electronic analysis.

Both primary and secondary literature were considered. Primary studies included RCTs and prospective as well as retrospective observational studies. Eligible studies met the following criteria: (1) comparison of ADSs vs. SD in patients with RVF; (2) participants aged over 18 years; and (3) reported data on survival. Secondary sources included systematic reviews and meta-analyses assessing the efficacy of ADSs in RVF, which were evaluated to provide a broader context and synthesis of existing evidence.

## 3. Main Text

### 3.1. Defibrillation Techniques: Rationale and Technical Considerations

**Figure 1:**
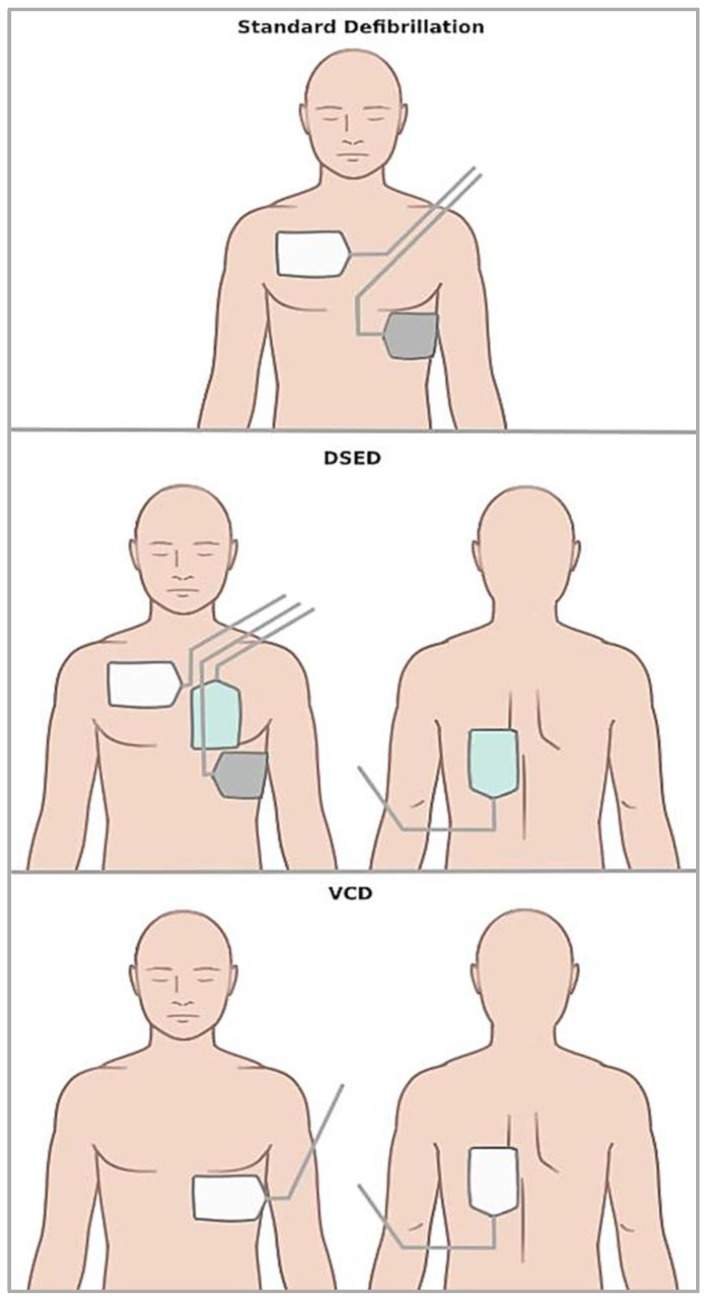
Scheme illustrating pad placement in the different defibrillation strategies.

DSED is the simultaneous use of two sets of manual defibrillators in two different planes, i.e., anterior–lateral and anterior–posterior [10]. Instead, VCD consists of shifting defibrillation pads from the usual anterior–lateral position to the anterior–posterior one [11]. Figure 1 graphically shows pad placement in standard defibrillation, DSED, and VCD.

Various dual defibrillation (DD) techniques have been described, including short delays (1–2 s) between sequential shocks, precisely synchronized overlapping shocks, or simultaneous shocks delivered by a single operator [12]. DSED, a specific DD technique, has been in use since 1994. Initially studied in patients who developed VF during electrophysiological procedures, it demonstrated success in terminating arrhythmias on the first attempt [13]. This dual-shock approach increases the total energy delivered and may depolarize a larger mass of myocardium more homogeneously, thereby enhancing the likelihood of interrupting all re-entrant circuits simultaneously. Additionally, the rapid sequential shocks may alter the transmembrane potential gradients more effectively than a single shock, helping to overcome localized zones of myocardial refractoriness with optimized current vectors [1,13]. VCD is another alternative approach for RVF. In SD, electrode pads are placed in an anterior–lateral position, directing current along a typical vector. Changing the position of the pads alters the direction and depth of the electrical current, potentially increasing the likelihood of capturing critical myocardial tissue that was previously missed. Pathophysiologically, this adjustment may help achieve more uniform myocardial depolarization by engaging areas of the heart that are less affected by the original vector, especially the posterior and septal regions. The new current pathway may also reduce areas of electrical shielding or anisotropic conduction that sustain fibrillatory wavefronts [1,11]. While both DSED and VCD aim to overcome the limitations of standard defibrillation by modifying the electrical vector, DSED may offer additional mechanistic advantages. In particular, the delivery of two high-energy shocks in rapid succession or simultaneously may result in broader and more homogeneous myocardial depolarization, the increased disruption of re-entrant circuits, and enhanced transmembrane potential shifts. These combined effects could explain the superior clinical outcomes observed with DSED in terms of ROSC and neurologically intact survival [1].

### 3.2. Evidence Overview

This narrative review synthesizes the current evidence on ADSs for the treatment of RVF. Early studies, mostly published before 2022, were dominated by retrospective observational designs [12,14,15,16,17], with only a few prospective investigations [18]. Notably, only one study focused specifically on VCD [14], while the others primarily examined DSED [19]. DSED was generally applied after three failed conventional defibrillations [12,16,17,18]. An exception is represented by the study by Emmerson et al. [15], in which DSED was implemented after six shocks (three in antero-lateral and three in antero-posterior positions). The technique involved either simultaneous [16,17] or rapid sequential shocks [12,15] using two defibrillators operated by a single provider. Energy settings varied: most studies used 200 J per defibrillator [12,17], while others employed a maximum energy of 360 J per device (delivered either in rapid succession [15] or simultaneously [16], for a total of 720 J). Despite these variations, DSED did not demonstrate consistent benefits over standard defibrillation in terms of ROSC [12,15,16,17,18], survival to hospital admission [12,15,16,18] or discharge [12,15,16,17,18], or neurologically intact survival [12,17]. Only the small study by Kim et al. reported an association between DSED and increased survival to hospital admission; however, this finding was not confirmed for survival to discharge or favorable neurological outcomes. Conversely, another study found DSED to be associated with lower odds of ROSC, possibly due to the excessively high energy used. Moreover, only one study specifically identified and analyzed the subgroup of patients with true RVF, without finding any difference in outcomes among defibrillation strategies [12]; all other studies relied on operative definitions and did not distinguish between refractory and recurrent VF [15,16,17,18]. Additionally, one descriptive study focused specifically on VCD and suggested a potential benefit in survival to hospital discharge, although the small sample size warrants cautious interpretation and further investigation [14]. As described, these studies exhibited considerable heterogeneity in design and defibrillation protocols, which limits their generalizability. Two metanalyses synthetizing the evidence from these studies failed to demonstrate a consistent survival benefit of ADSs over SD, highlighting the need for higher-quality evidence [20,21].

The RCT by Cheskes et al. represented a pivotal advancement, showing that both DSED and VCD were associated with significantly improved survival to hospital discharge in patients with RVF [1]. Regarding secondary outcomes, DSED was superior in achieving the termination of VF, the return of spontaneous circulation (ROSC), and favorable neurological outcomes compared to SD. VCD, on the other hand, showed a greater effect on VF termination alone. The study was distinguished by its robust design and large sample size. However, it was prematurely terminated by the ethics committee due to pandemic-related disruptions, limiting its statistical power. The authors also reported a relatively low fragility index: the survival benefit would have lost significance if nine fewer patients in the DSED group or one fewer in the VCD group had survived. Despite this, the trial marked a turning point in evaluating ADSs and was conceived as a continuation of the earlier DOSE-VF trial [22].

A substudy of the trial demonstrated that these improved outcomes occurred independently of in-hospital interventions, such as coronary angiography or percutaneous coronary intervention (PCI), emphasizing the potential prehospital value of ADSs [23]. In addition, the RCT adopted a pragmatic definition of RVF as VF persisting after three shocks, but did not differentiate whether the first three shocks addressed shock-refractory or recurrent VF. A secondary analysis was conducted to categorize patients, accordingly, based on the pre-randomization phase [19]. Post-randomization outcomes were then analyzed to evaluate the effects of SD, VCD, or DSED on patient outcomes. The secondary analysis demonstrated higher survival rates in patients with recurrent VF compared to those with shock-refractory VF. In this analysis, DSED emerged as the superior defibrillation strategy, regardless of whether the preceding VF was shock-refractory or recurrent. In contrast, VCD was not associated with improved survival in either group [19]. Additionally, another secondary analysis demonstrated that both DSED and VCD significantly reduced VF duration compared to SD [24]. After the publication of the most recent data by Cheskes et al. [1], several systematic reviews and meta-analyses emerged, aiming to contextualize these findings within the broader body of literature [25,26]. These studies concluded there being no association of DSED or VCD with better survival to hospital discharge. However, these findings must be interpreted cautiously due to several limitations. First, only a limited number of studies met the inclusion criteria. Second, the validity of these pooled estimates is limited by the combination of one high-quality RCT with numerous low-quality observational studies, raising concerns about heterogeneity and bias. These discrepancies may be partly explained by the lack of standardized protocols across earlier studies, including differences in shock timing (simultaneous vs. sequential), energy settings, and the timing of DSED application. In contrast, the DOSE-VF trial applied a uniform escalation strategy and predefined criteria, which likely contributed to the observed more consistent and favorable outcomes.

Beyond clinical outcomes, a critical limitation in the current literature is the lack of equity-focused analyses. No study to date has conducted sex- or gender-disaggregated analyses, despite the 2016 SAGER (Sex and Gender Equity in Research) guidelines [27]. When pooling data from the available primary literature, women comprise only ~19% of the study population, although they account for up to 42% of OHCA cases [28,29]. This under-representation is consistent with broader trends observed in cardiovascular and acute care research, where female patients are often excluded or inadequately analyzed [30,31,32,33]. Despite this significative lack of data, sex-based differences in CA management and survival have been reported. Indeed, it was observed that all survival outcomes were higher in males experiencing OHCA than in females [34,35]. These differences were partly explained by different trends in initial shockable rhythm, the provision of in-hospital procedures [34], age [35], and disposable outcomes [35]. Ethnicity is another critical but under-reported variable; only one study provides detailed participant data on it [18]. Existing epidemiological data indicate that African Americans exhibit lower survival rates than Whites, which is likely attributable to a lower incidence of witnessed cardiac arrests and resuscitation performed by rescuers, in addition to a less favorable presentation rate [36]. The absence of sex- and ethnicity-specific data significantly limits the generalizability of the current findings and highlights the need for inclusive trial designs and disaggregated reporting in future research.

### 3.3. Strengths and Limitations of the Current Evidence

The literature on ADSs has evolved significantly in recent years, offering valuable insights into a previously underexplored area of cardiac arrest management. One of the key strengths of this emerging body of work is the growing recognition of RVF as a distinct and clinically important phenotype, prompting targeted investigations. The publication of the RCT by Cheskes et al. [1] represented a watershed moment, introducing high-quality randomized evidence into a field previously dominated by observational studies.

However, several critical limitations restrict the interpretability and applicability of the current evidence. Firstly, most studies are affected by small sample sizes [14,17,18] and heterogeneous protocols for ADS application, including differences in shock energy and timing [12,14,15,16,17,18]. Indeed, there is currently no consensus on core aspects such as pad placement, the sequence and timing of shocks, or coordination between EMS providers (i.e., if single or double operators). This operational variability limits reproducibility and may partly explain the heterogeneity in outcomes. Several observational studies [14,15,16,17,18] did not distinguish between shock-refractory and recurrent VF, which may represent physiologically distinct entities with different responses to defibrillation. Methodological inconsistencies, combined with the absence of blinding and the high risk of bias in most available studies, undermine the internal as well as external validity of the findings and complicate cross-study comparisons.

The fact that most of the evidence is prehospital in origin also limits the understanding of ADSs’ utility in in-hospital cardiac arrest (IHCA) or in healthcare systems with differing levels of EMS organization, training, and technology.

The generalizability of existing findings is further compromised by narrow demographic representation. As detailed above, sex- and ethnicity-based analyses are lacking or underpowered, precluding any meaningful conclusions regarding differential treatment effects in diverse populations. The systematic exclusion or under-representation of women and ethnic minorities continues to hinder efforts toward equitable resuscitation science. In addition to under-representation, sex-based physiological differences (such as myocardial mass, conduction velocity, and autonomic tone) may influence defibrillation thresholds and current distribution during ADSs. These factors could potentially modulate the efficacy of DSED and VCD, underscoring the need for sex-disaggregated analyses in future trials.

Despite the limitations of the observational studies highlighted above, and given the presence of one well-conducted RCT, the overall level of evidence could be classified as B-R (i.e., moderate quality from one RCT).

### 3.4. Practical Implications for Clinical Management

In the setting of OHCA with RVF, clinicians must reach a critical decision. The persistence of VF at this stage is associated with extremely poor outcomes unless circulation is rapidly restored [37]. While epinephrine and ongoing high-quality CPR remain essential, defibrillation remains the only definitive treatment for VF [8,9]. Thus, reconsidering the method of defibrillation becomes a reasonable and time-sensitive intervention. It is important to acknowledge that the availability of dual defibrillators and trained personnel may be limited in low- to middle-income countries. In such settings, VCD may represent a more feasible escalation strategy, given its lower logistical demands. Future implementation studies should explore the cost-effectiveness, training requirements, and workflow integration of alternative defibrillation strategies across different healthcare systems.

### 3.5. Escalation Strategy

From a practical standpoint, it is reasonable for the resuscitation team to actively consider implementing an ADS after three failed standard shocks. This requires that RVF is not only recognized promptly but also that a predetermined escalation plan exists. The absence of such a plan often leads to the repeated delivery of ineffective standard shocks, which may contribute to electrical injury and progressive myocardial dysfunction [38].

### 3.6. Decision Making: DSED vs. VCD

Among the available strategies, DSED appears to offer the most promising signal for improved outcomes. However, DSED is not plug-and-play. For successful implementation, the following conditions should be met: (i) the availability of two defibrillators and both must be immediately accessible at the scene; (ii) pre-assigned team roles, i.e., one operator must coordinate both shocks, ensuring near-simultaneous delivery without delay; and (iii) pad positioning familiarity; therefore, the team must be trained to rapidly apply the second set of pads in the anteroposterior position, ideally within the ongoing cycle of CPR.

If DSED is not feasible (either because a second defibrillator is not available or because team familiarity is limited), the choice between VCD and SD becomes more complex, especially considering the fact that VCD is supported by less scientific evidence [1,14]. However, VCD is supported by a high-quality RCT [1] and it is easier to implement in most settings because (i) it requires only one defibrillator; (ii) it avoids potential confusion or misfiring associated with dual-shock coordination; and (iii) it can be adopted by teams with minimal additional training, provided the pad change is executed efficiently during CPR. Given the technical demands of DSED, a tiered approach to implementation may be appropriate. Basic EMS teams could be trained to apply VCD, while advanced resuscitation teams may be designated and equipped for DSED delivery. This model allows for the integration of ADSs based on local resources and team capabilities.

### 3.7. Timing and Integration into ACLS

Regardless of the defibrillation strategy selected, it is essential that escalation occurs promptly, ideally immediately after the third standard shock has failed and VF persists. Delaying this transition while continuing with repeated standard shocks may diminish the likelihood of a successful outcome. Therefore, ADSs should be fully embedded within local ACLS protocols [8,9]. This includes ensuring that field personnel are thoroughly trained in both the indications and execution of these strategies, and that standard operating procedures clearly define when and how to escalate.

### 3.8. Avoiding Disruption of Core Resuscitation Quality

It is essential to emphasize that the introduction of DSED or VCD must not disrupt the continuity of chest compressions, nor delay drug administration, airway management, or transport decisions. Resuscitation team leaders must ensure that any escalation in defibrillation strategy is carried out within the framework of uninterrupted, high-quality ACLS [8,9].

### 3.9. In-Hospital Applications

While current evidence is drawn mostly from prehospital environments [1,12,14,15,16,17,18], hospitals (especially emergency departments and intensive care units, ICUs) are well positioned to explore the use of ADSs. In these settings, defibrillators and personnel are typically more readily available, and protocols can be standardized with institutional support. Training advanced response teams in DSED or VCD can represent a logical step forward for patients with in-hospital RVF, even if direct evidence is still lacking.

**Figure 2:**
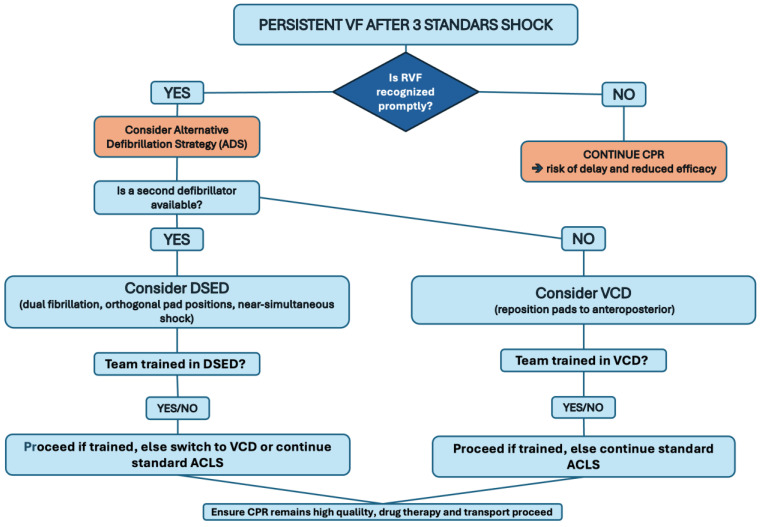
Proposed practical approach to RVF.

To support the practical application of these principles, we developed a step-by-step decision-making algorithm in the form of a flow chart, which outlines the recommended actions following a third unsuccessful shock (Figure 2). We acknowledge that this flow diagram has not been formally validated; however, it may serve as a useful tool to support clinical decision making in the management of patients with RVF.

### 3.10. Future Directions

Despite promising early results, the field of ADSs remains in a formative stage, with several key areas requiring further exploration to optimize outcomes in patients with RVF. One of the most pressing needs in the field is the completion of large-scale, multicenter RCTs to definitively establish the comparative effectiveness of DSED and VCD across varied clinical environments. These trials should be complemented using standardized protocols, clearly defined outcome measures, and the inclusion of diverse patient populations to enhance generalizability. Moreover, evaluating these defibrillation strategies in in-hospital settings, where their implementation may be more feasible and controlled, represents an important avenue for future research.

Another key priority is the refinement of patient selection criteria. Not all patients with refractory RVF may benefit equally from ADSs [19]. Identifying clinical, electrocardiographic, or even genetic markers that predict a favorable response could pave the way for more-personalized resuscitation approaches. Concurrently, mechanistic studies are warranted to elucidate how factors such as shock vector orientation, pad positioning, and energy delivery interact with the myocardial substrate during prolonged VF. In this context, real-time thoracic impedance monitoring may offer a novel tool with which to guide energy titration and pad positioning during ADSs. Impedance-guided defibrillation could help personalize shock delivery and warrants investigation as part of future precision resuscitation strategies. In parallel, preclinical studies using optical mapping, computational modeling, or animal models could provide valuable mechanistic insights into how DSED and VCD interact with myocardial substrate and disrupt re-entrant circuits. These approaches may help clarify the biophysical basis of ADS efficacy and guide the optimization of energy delivery and pad positioning.

Another critical frontier lies in the integration of ADSs into real-time clinical decision making. The use of machine learning algorithms embedded within defibrillators or EMS dispatch systems could assist in the early recognition of RVF and prompt timely escalation to ADSs [39]. These technologies, combined with wearable or implantable sensors, may one day enable the pre-emptive identification of patients at high risk for shock-refractory arrest.

The successful adoption of ADSs will depend on robust training frameworks, standardized protocols, and system-wide quality improvement initiatives. Simulation-based education, real-time feedback tools, and post-event debriefing may enhance team performance and adherence to escalation algorithms. Furthermore, implementation science will be essential to bridge the gap between evidence and practice, particularly in resource-limited settings where access to dual defibrillators or advanced training may be constrained.

## 4. Conclusions

RVF represents one of the most challenging scenarios in cardiac arrest management, with persistently poor outcomes despite adherence to current ACLS protocols. In this context, ADSs have emerged as promising interventions aimed at improving defibrillation success and survival. While a pivotal RCT suggested potential benefits (especially for DSED), current evidence remains limited mostly by the predominance of heterogeneous and low-quality observational studies, methodological inconsistencies, the under-representation of women and ethnic minorities, and a lack of standardized implementation protocols.

The integration of ADSs into clinical practice requires not only further validation through large-scale, multicenter trials, but also a paradigm shift in how resuscitation teams approach shock-refractory rhythms. The timely recognition of RVF, structured escalation algorithms, and targeted training are essential to ensure that these strategies are applied effectively and without compromising the quality of core resuscitation efforts. Moreover, future research must address critical gaps in equity and representation, including sex- and ethnicity-based analyses, to ensure that emerging interventions benefit all patient populations. Whether ADSs will become standard components of ACLS or remain advanced escalation tools will depend on future evidence and system capacity. A tiered approach (where VCD is broadly implemented and DSED reserved for specialized teams) may represent a pragmatic step forward.

Ultimately, ADSs should not be viewed as isolated techniques but as components of a broader, evolving framework of precision resuscitation. Their successful adoption will depend on a combination of robust evidence, operational readiness, and system-wide commitment to continuous improvement. As the field advances, emergency physicians will play a pivotal role in translating innovation into practice, ensuring that patients with RVF receive the most effective and timely care possible.

## Data Availability

This research does not directly involve patients; hence, no dataset has been created.

## Supplementary Materials

The following supporting information can be downloaded at: https://www.mdpi.com/article/10.3390/jcm14145016/s1, Supplementary Table S1. Complete literature search strategy.

## Abbreviations

ACLS	Advanced cardiovascular life support
ADS	Alternative defibrillation strategies
AHA	American Heart Association
CA	Cardiac arrest
CPR	Cardiopulmonary resuscitation
DSED	Double sequential external defibrillation
EMS	Emergency medical service
ERC	European Resuscitation Council
ICU	Intensive care unit
IHCA	In-hospital cardiac arrest
OHCA	Out-of-hospital cardiac arrest
PCI	Percutaneous coronary intervention
PEA	Pulseless electric activity
pVT	Pulseless ventricular tachycardia
RCT	Randomized controlled trial
ROSC	Return of spontaneous circulation
RVF	Refractory ventricular fibrillation
SD	Standard defibrillation
SGBA	Sex- and gender-based analysis
VCD	Vector-change defibrillation
VF	Ventricular fibrillation
