# Supplementary material for: Beyond Standard Shocks: A Critical Review of Alternative Defibrillation Strategies in Refractory Ventricular Fibrillation
Source: J Clin Med. 2025 Jul 15;14(14):5016. doi: 10.3390/jcm14145016 (PMC12295630; doi:10.3390/jcm14145016)
Supplement: Supplementary file 1 [file jcm-14-05016-s001.zip › jcm-3748286-Supplementary Table S1.pdf]

Journal of Clinical Medicine

MDPI

Supplementary Materials:

Supplementary Table S1. Complete literature search strategy.

Search strategy		
"Double-sequential external defibrillation"	**AND**	"Refractory ventricular fibrillation"<br>"Cardiac arrest"<br>"Ventricular fibrillation"<br>"VF"<br>"Pulseless ventricular tachycardia"<br>"pVT"
"Dual-sequential external defibrillation"	**AND**	"Refractory ventricular fibrillation"<br>"Cardiac arrest"<br>"Ventricular fibrillation"<br>"VF"<br>"Pulseless ventricular tachycardia"<br>"pVT"
"DSED"	**AND**	"Refractory ventricular fibrillation"<br>"Cardiac arrest"<br>"Ventricular fibrillation"<br>"VF"<br>"Pulseless ventricular tachycardia"<br>"pVT"
"Vector-change defibrillation"	**AND**	"Refractory ventricular fibrillation"<br>"Cardiac arrest"<br>"Ventricular fibrillation"<br>"VF"<br>"Pulseless ventricular tachycardia"<br>"pVT"
"VCD"	**AND**	"Refractory ventricular fibrillation"<br>"Cardiac arrest"<br>"Ventricular fibrillation"<br>"VF"<br>"Pulseless ventricular tachycardia"<br>"pVT"
"Defibrillation strategies"	**AND**	"Refractory ventricular fibrillation"<br>"Cardiac arrest"<br>"Ventricular fibrillation"<br>"VF"<br>"Pulseless ventricular tachycardia"<br>"pVT"
